# The effect of infliximab in patients with chronic low back pain and Modic changes (the BackToBasic study): study protocol of a randomized, double blind, placebo-controlled, multicenter trial

**DOI:** 10.1186/s12891-020-03720-5

**Published:** 2020-10-21

**Authors:** Elisabeth Gjefsen, Lars Christian Haugli Bråten, Guro Løvik Goll, Monica Wigemyr, Nils Bolstad, Morten Valberg, Elina Iordanova Schistad, Gunn Hege Marchand, Fredrik Granviken, Kaja Kristine Selmer, Anne Froholdt, Anne Julsrud Haugen, Magnhild Hammersland Dagestad, Nils Vetti, Gunnstein Bakland, Benedicte Alexandra Lie, Espen A. Haavardsholm, Aksel Thuv Nilsen, Thor Einar Holmgard, Thomas Istvan Kadar, Tore Kvien, Jan Sture Skouen, Lars Grøvle, Jens Ivar Brox, Ansgar Espeland, Kjersti Storheim, John Anker Zwart

**Affiliations:** 1 Research and Communication Unit for Musculoskeletal Health (FORMI), Oslo University Hospital HF, Ulleval, Bygg 37b, P.O. Box 4956 Nydalen, 0424 Oslo, Norway; 2 Faculty of Medicine, University of Oslo, P.O. Box 1072 Blindern, 0316 Oslo, Norway; 3 Department of Rheumatology, Diakonhjemmet Hospital, Box 23 Vinderen, 0319 Oslo, Norway; 4 Department of Research and Innovation, Division of Clinical Neuroscience, Oslo University Hospital, P.O. Box 4956 Nydalen, 0424 Oslo, Norway; 5 Department of Medical Biochemistry, Oslo University Hospital, Radiumhospitalet, Box 4953 Nydalen, 0424 Oslo, Norway; 6 Oslo Centre for Biostatistics and Epidemiology, Oslo University Hospital, Sogn Arena 3.etg, P.O.Box 4950 Nydalen, Oslo, Norway; 7 Department of Physical Medicine and Rehabilitation, Oslo University Hospital HF, P.O. Box 4956 Nydalen, 0424 Oslo, Norway; 8 Department of Physical Medicine and Rehabilitation, St. Olavs Hospital, Trondheim University Hospital, P.O. Box 3250 Torgarden, NO-7006 Trondheim, Norway; 9 Department of Neuromedicine and Movement Science, Faculty of Medicine and Health Sciences, Norwegian University of Science and Technology, Trondheim, 7491 Norway; 10 Department of Public Health and Nursing, Faculty of Medicine and Health Sciences, Norwegian University of Science and Technology, Trondheim, 7491 Norway; 11 National Centre for Epilepsy, Oslo University Hospital, Oslo, Norway; 12 Department of Physical Medicine and Rehabilitation, Drammen Hospital, Vestre Viken Hospital Trust Drammen, P.O. Box 800, 3004 Drammen, Norway; 13 Department of Rheumatology, Østfold Hospital Trust, P.O. Box 300, 1714 Grålum Moss, Norway; 14 Department of Radiology, Haukeland University Hospital, Jonas Liesvei 65, 5021 Bergen, Norway; 15 Department of Clinical Medicine, University of Bergen, P.O. Box 7804, 5020 Bergen, Norway; 16 Department of Rheumatology, University Hospital of North Norway, P.O. Box 100, 9038 Tromsø, Norway; 17 Department of Medical Genetics, University of Oslo and Oslo University Hospital, P.O. Box 4956 Nydalen, 0424 Oslo, Norway; 18 Norwegian Back Pain Association, P.O.Box 9612 Fjellhagen, 3065 Drammen, Norway; 19 Department of Physical Medicine and Rehabilitation, Haukeland University Hospital, Helse Bergen HF, Box 1, 5021 Bergen, Norway; 20 Department of Global Public Health and Primary Care, University of Bergen, Kalfarveien 31, 5018 Bergen, Norway; 21 Department of Physiotherapy, Oslo Metropolitan University, P.O. Box 4 St. Olavs plass, NO-0130 Oslo, Norway

**Keywords:** Low back pain, Modic changes, Inflammation, Clinical trial, TNF- α inhibitor, Infliximab, Randomized controlled trial

## Abstract

**Background:**

Low back pain is common and a significant number of patients experience chronic low back pain. Current treatment options offer small to moderate effects. Patients with vertebral bone marrow lesions visualized as Modic changes on magnetic resonance imaging may represent a subgroup within the low back pain population. There is evidence for inflammatory mediators being involved in development of Modic changes; hence, suppression of inflammation could be a treatment strategy for these patients. This study examines the effect of anti-inflammatory treatment with the TNF-α inhibitor infliximab in patients with chronic low back pain and Modic changes.

**Methods/design:**

The BackToBasic trial is a multicenter, double blind, randomized controlled trial conducted at six hospitals in Norway, comparing intravenous infusions with infliximab with placebo. One hundred twenty-six patients aged 18–65 with chronic low back pain and type 1 Modic changes will be recruited from secondary care outpatients’ clinics. The primary outcome is back pain-specific disability at day 154 (5 months). The study is designed to detect a difference in change of 10 (SD 18) in the Oswestry Disability Index at day 154/ 5 months. The study also aims to refine MRI-assessment, investigate safety and cost-effectiveness and explore the underlying biological mechanisms of Modic changes.

**Discussion:**

Finding treatments that target underlying mechanisms could pose new treatment options for patients with low back pain. Suppression of inflammation could be a treatment strategy for patients with low back pain and Modic changes. This paper presents the design of the BackToBasic study, where we will assess the effect of an anti-inflammatory treatment versus placebo in patients with chronic low back pain and type 1 Modic changes.

The study is registered at ClinicalTrials.gov under the identifier NCT03704363. The EudraCT Number: 2017–004861-29.

## Background

Low back pain (LBP) is the leading cause of disability worldwide [1]. The condition is very common, affecting all age groups and the costs for patients and society are immense [2]. About 10% of patients with LBP develop chronic LBP (cLBP). The vast majority of patients (80–90%) are classified as having non-specific LBP, [3] and treatment focus on reducing symptoms [4]. Unfortunately, these treatment options offer only small to moderate effects [2]. Researchers therefore attempt to identify subgroups within the non-specific LBP group that are likely to respond favorably to specific treatment [5, 6]. Patients with vertebral bone marrow lesions visualized as Modic changes (MCs) on magnetic resonance imaging (MRI) have been suggested to represent such a subgroup [7].

MCs are defined as type 1, 2 and 3 based on T1- and T2 weighted MRI [8, 9]. Type 1 (oedema type) is hypo-intense on T1- and hyper-intense on T2- weighted MRI, type 2 (fatty type) is hyper-intense on T1- and T2- weighted MRI, and type 3 (sclerotic type) is hypo-intense on T1- and T2- weighted MRI. Combined types are common. MC types can change over time, and the different types are thought to reflect a common underlying process [8, 10].

Several studies have reported a possible association between cLBP and MCs [11]. The association is not consistent, [12] but possibly more evident for type 1 than type 2 MCs [13–15]. Despite clinical experience that MCs can be painful; the etiology is unknown, though an infectious, mechanical, or autoimmune pathogenesis is hypothesized [16–20]. A Danish randomized controlled trial (RCT) from 2013 reported that 100 days of antibiotic treatment was substantially more effective than placebo in patients with LBP and type 1 MCs [21]. These results were not confirmed in a recently published Norwegian RCT [22]. The diverging results underscore the need for further research to understand the underlying causes and significance of MCs.

The autoimmune theory suggests that MCs are secondary to a biomechanical degradation that causes a subsequent autoimmune response [7]. Animal models have shown that nucleus pulposus cells are immunogenic and may trigger an inflammatory cascade resulting in MCs [20]. Independent of what the true etiology of MCs is, there is evidence for inflammatory mediators being involved in painful disc degeneration and MCs [7, 23]. TNF-α is a pleiotropic pro-inflammatory cytokine that is found to be expressed in MCs, along with IL-1 β, IL-6 and IL-8 [24]. Also, Othori et al. found significantly more TNF-immunoreactive cells in MCs compared with patients without MCs [25]. Clinical trials suppressing the inflammatory response by giving steroid injections or TNF-α inhibitors in patients with acute LBP and sciatica have been conducted [26–29]. In the Korhonen study, a subgroup with concomitant MCs had a possible beneficial effect of TNF-α inhibitors [29]. Thus, suppression of inflammation emerges as a possible treatment strategy for patients with cLBP and MCs.

This article details the protocol of the BackToBasic study, which aims to assess the effect of treatment with the TNF-α inhibitor infliximab on disease-specific disability in patients with cLBP and type 1 MCs.

The study will also explore underlying biological mechanisms of MCs by studying potential biomarkers, investigate the gut microbiome, genetic susceptibility and epigenetics, evaluate cost-effectiveness, and refine MRI assessment for cLBP with MCs.

## Methods

### Overview of study design

The BackToBasic study is a multicenter, randomized, placebo-controlled, double-blind phase III trial of infliximab in patients with cLBP and type 1 MCs. The schedule for enrolment, interventions and assessments are given in Table 1.

**Table 1:** Schedule of enrolment, interventions and assessments (SPIRIT)

TIMEPOINT	STUDY PERIOD						
	Screening	Baseline/ treatment	Treatment period			End of study	Safety registration
	***Within 6 weeks before baseline***	Day 0	***Day 14***	***Day 42***	***Day 98***	***Day 154***	***Day 278***
**ENROLMENT:**							
**Eligibility screen**	X						
**Informed consent**	X						
**Medical history**	X						
**MRI-scan**^a^	X					X	
**Chest X-ray**	X						
**Urine pregnancy test**^b^	X	X		X	X	X	
**Blood samples (hematology, clinical chemistry, CRP)**^c^	X	X	X	X	X	X	
**TB, Hep C, Hep B-screening**	X						
**Allocation**		X					
**INTERVENTION:**							
**Infliximab**		X	X	X	X		
**Placebo**		X	X	X	X		
**ASSESSMENTS:**							
**Background data**^d^	X	X					
**Clinical safety evaluation**^e^	X	X	X	X	X	X	
**Clinical pain/neuro evaluation**^f^	X						
**Blood samples for drug concentrations and antibodies**^g^		X	X	X	X	X	
**Blood samples for biobank**		X			X	X	
**Adverse events**			X	X	X	X	X
**Primary outcome**^h^		X	X	X	X		X
**Pain monitoring**^i^		X	X	X	X	X	X
**Concomitant medication**	X	X	X	X	X	X	X
**Co-interventions (non-pharm)**	X	X	X	X	X	X	X
**Sick listing**		X			X	X	X
**Questionnaires**^j^		X			X	X	
**Compliance**^k^		X	X	X	X		

^a^Baseline MRI according to the study protocol can be maximum 4 weeks old when treatment starts. A follow-up MRI is taken between 6 and 7 months after treatment start (i.e. 7 to 8 months after baseline MRI).

^b^Urine pregnancy test will be performed at screening and monthly from treatment initiation until 9 months. Results will be enquired with telephone follow up.

^c^Haematological parameters (hemoglobin, haematocrit (hct), erythrocytes, white blood cells with differentials, platelet counts), Clinical chemistry (AST and/or ALT, ALP, albumine, creatinine, random glucose, potassium, sodium) and CRP (SLV-imposed). Random glucose is for further safety monitoring (self-imposed)

^d^Baseline data

^e^Weight, blood pressure, pulse, auscultation of hearth and lunges, GI and neurological examination

^f^Pain provocation tests, neurological tests

^g^Antibodies to infliximab

^h^ODI

^i^Pain-monitoring (LBP intensity) weekly during follow-up period

^j^EQ 5D-5L,RMDQ, Patients’satisfaction, global perceived effect, symptom specific well-being, leg pain intensity, hours with LBP last 4 weeks

^k^Number of completed intravenous infusions with the IMP

### Study population and recruitment

**Fig. 1:**
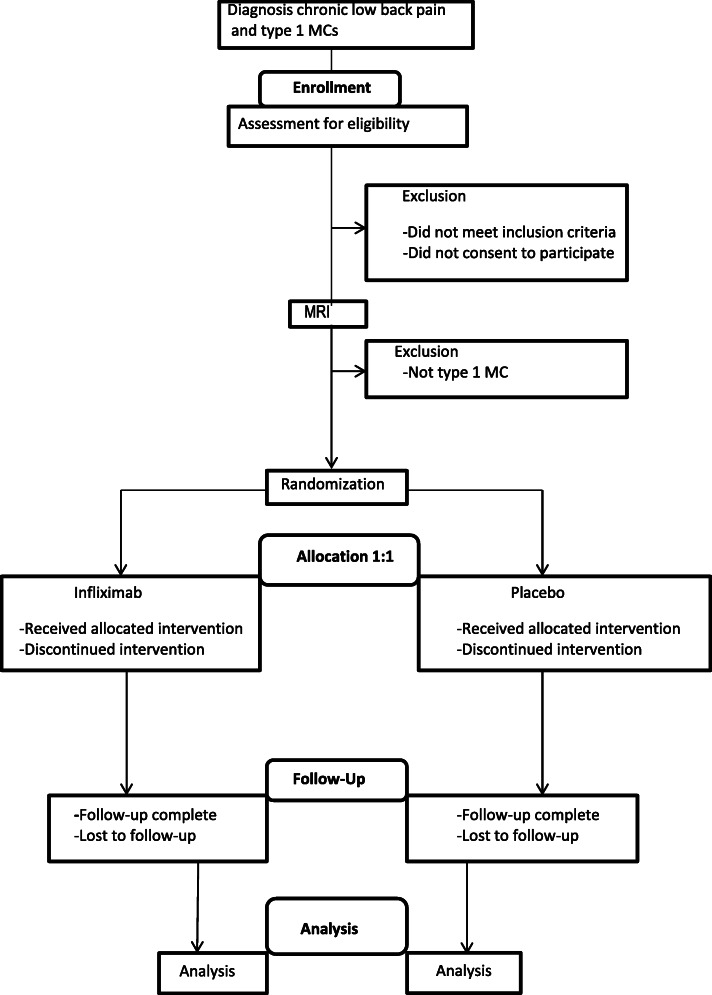
Flow-chart of the BackToBasic trial

The flow of patients in the BackToBasic study is illustrated in Fig. 1. We plan to include and randomize 126 patients with cLBP and type 1 MCs. The first patient was included December 2018. Patients referred to secondary care outpatient clinics due to cLBP are screened for eligibility at the six participating hospitals in all health regions in Norway. (Oslo University Hospital, Ullevål; Haukeland University Hospital, Bergen; St. Olavs Hospital, Trondheim; University Hospital of North Norway, Tromsø; Østfold Hospital Trust, Moss; Vestre Viken Hospital trust, Drammen). Recruiting clinicians screen eligible patients for inclusion and exclusion criteria, and refer for a standardized study-specific baseline 1.5 T MRI examination to confirm and characterize the MCs. The MRI scans are de-identified and independently evaluated by two study radiologists.

To be included in the trial participants must fulfill the following inclusion criteria:

- Age between 18 and 65 years
- LBP of > 50% of days for > 6 months duration in the area below the 12th rib and above the gluteal folds with a Numerical Rating Scale (NRS) pain intensity score of ≥ 5 (mean of three 0–10 NRS scales: current LBP, the worst LBP within the last 2 weeks, and the usual/mean LBP within the last 2 weeks) and/or Oswestry Disability Index (ODI-score) ≥25
- MC of craniocaudal size ≥10% of vertebral height and of primary or secondary type 1 in the vertebral body at a level of the lumbar spine (superior or inferior endplate, Th12-S1)
- Negative pregnancy test
- Signed informed consent obtained and documented according to ICH GCP, and national/local regulations

The patients will be excluded if there is a specific diagnosis that may explain their low back symptoms (e.g. tumour, fracture, spondyloarthritis, infection, spinal stenosis), former low back surgery (L1 – S1) for other reasons than disc herniation or decompression, and also if surgery for disc herniation or decompression has been carried out within the last 12 months prior to inclusion. Patients who regularly use opioids with the exception of codeine and tramadol are not eligible. Further exclusion criteria include infections, pregnancy, diabetes, immunodeficiency or the use of immunosuppressive medication. A full list of exclusion criteria is provided in Table 2.

**Table 2:** Full list of exclusion criteria

Exclusion criteria:	
• Fever or ongoing infection	
• Allergy or hypersensitivity against any products of the medication	
• Previous infliximab treatment	
• Any serious adverse events with other immunosuppressive treatment (including cytostatics, antibodies, drugs acting on immunophilins, Interferons, mycophenolate and any other DMARDs)	
• Any specific diagnosis that may explain patient’s low back symptoms (e.g. tumour, fracture, spondyloarthritis, infection, spinal stenosis).	
• Former low back surgery (L1 – S1) for other reasons than disc herniation or decompression (e.g. fusion, disc prosthesis).	
• Former surgery for disc herniation or decompression within the last 12 months	
• Any known rheumatic disease	
• Current pregnancy or lactation	
• For women of childbearing potential (WOCBP); inadequate birth control, pregnancy, and/or breastfeeding. WOCBP is defined as those who are fertile (with uterus, fallopian tubes and at least one intact functional ovary), following menarche and until becoming post-menopausal unless permanently sterile. Permanent sterilization methods include hysterectomy, bilateral salpingectomy and bilateral oophorectomy. Documentation of surgical procedure or physical examination is required for subjects who have had such an operation. Adequate contraception must be used by WOCBP during the entire intervention period and 6 months after the last administration of study drug, and includes oral, injected or implanted hormonal methods of contraception, placement of an intrauterine device or system, vasectomized partner or sexual abstinence.	
• Ongoing systemic glucocorticoid or other immunosuppressive treatments (see list above)	
• Regular use of opioids with the exception of codeine and tramadol	
• Other immunosuppressive treatment last year (see list above)	
• Active or latent (known or suspected) tuberculosis (all participants will be screened for latent tuberculosis)	
• Previous infection with Hepatitis B virus (HBV) (all participants will be screened for HBV-carrier state)	
• Live vaccination within the last 4 weeks or planned live vaccination during treatment period	
• Planned surgical procedure	
• Increased transaminases (ASAT/ALAT)	
• Ongoing or previous malignant disease at any time (i.e. skin cancer, cervical cancer etc.)	
• Known increased risk of malignant disease	
• Diabetes	
• Immunodeficiency (i.e. primary immunodeficiency diseases, human immunodeficiency virus/acquired immunodeficiency syndrome, splenectomy)	
• Heart failure (NYHA class III - IV)	
• Previous or ongoing psoriasis	
• Ulcerative colitis or Crohns disease	
• Existing or recent demyelination diseases (I.e. MS or Guillain-Barres)	
• Abnormal hemoglobin or abnormal platelet, leucocyte or neutrophil count	
• Not able to understand written and spoken Norwegian	
• Not able to complete treatment or follow-ups in the study (i.e. severe psychiatric disease, drug abuse or plans of moving address)	
• Contra indications for MRI (i.e. pacemaker, metal implants, claustrophobia)	
• Abnormal creatinine level	

### Data registration and monitoring

The web-based eCRF software solution, Viedoc™, (Pharma Consulting Group, Uppsala, Sweden) is used to collect study data. The Principal Investigator at each study center is responsible for assuring that data entered into the eCRF are complete, accurate and entered in a timely manner. The electronic signature of the investigator will attest the accuracy of the data in each eCRF. If any assessments are omitted, the reason will be noted on the eCRFs. Corrections will be recorded giving their reason. A complete list of authorized study personnel will be maintained during the study, and only authorized study personnel will be allowed to sign the eCRF. Protocol, protocol amendments, investigator’s brochure and all study-related documents have been reviewed by an institutional review board and a Good Clinical Practice (GCP) medical monitor. All participating centers will be monitored during and after the trial in order to ensure compliance with GCP, the protocol and all other applicable regulations. The monitoring is conducted by the Clinical Trial Unit, Oslo University Hospital; Clinical Research Department, St. Olavs Hospital, Trondheim; Centre for Quality Improvement and Development, Research and Innovation, Research & Development, Haukeland University Hospital; Clinical Research Department, Centre for Quality Improvement and Development, University Hospital of North Norway,

### Data collection

Data will be collected and entered in Viedoc™ at screening, baseline, during the treatment period and at day 154 /5 months and day 278/9 months after the first treatment, regardless of patients’ compliance to the study protocol, following the Recommendations for Interventional Trials (SPIRIT).

During the trial, patients fill in patient reported outcome measures (PROMs) via ViedocMe (eCRF). Table 3 shows the full set of PROMs and time schedule. Patients enter data via a smartphone, tablet or PC using a personal username and password. If the forms are not filled out in time, they will receive two automatically generated SMS or email-reminders. For participants unable to use the ViedocMe eCRF, a paper version will be available, and study personnel will transfer the data into the eCRF.

**Table 3:** Patient reported outcome measures

Outcome measures	Timeline
– Oswestry Disability Index (ODI) 2.0, range 0–100 (Primary outcome)	Day 0, 28, 56, 91, 120, 154(5 months) and 278 (9 months)
– Low back pain intensity (mean of three Numeric Rating Scales (NRSs, range 0–10); current LBP, the worst LBP within the last 2 weeks, and usual/mean LBP within the last 2 weeks (for weekly reports during the intervention period; the wording “last 2 weeks” will be replaced by “the last week”)	Every week during treatment period and at day 154(5 months) and 278 (9 months)
– Roland and Morris Disability Questionnaire (RMDQ), range 0–24	Day 0, 56, 154(5 months) and 278 (9 months)
– Leg pain intensity (NRSs, range 0–10) last week	Day 0, 56, 154(5 months) and 278 (9 months)
– Hours with LBP during the last 4 weeks	Day 0, 56, 154(5 months) and 278 (9 months)
– Symptom-specific well-being	Day 0, 56, 154(5 months) and 278 (9 months)
– Days with sick leave	Day 0, 28, 56, 91, 120, 154(5 months) and 278 (9 months)
– Co-interventions	Day 0, 28, 56, 91, 120, 154(5 months) and 278 (9 months)
– Concomitant medication	Day 0, 28, 56, 91, 120, 154(5 months) and 278 (9 months)
– Patients’ satisfaction	Day 0, 56, 154(5 months) and 278 (9 months)
– Global perceived effect	Day 0, 56, 154(5 months) and 278 (9 months)
– EQ. 5D-5L	Day 0, 56, 154(5 months) and 278 (9 months)
– Emotional distress (Hopkins Symptom Checklist–25)	Reported at baseline
– Fear-avoidance beliefs Questionnaire (FABQ)	Reported at baseline
– Subjective health complaints (SHC)	Reported at baseline
– Background information	Reported at baseline
– Perceived treatment	Day 7, 56, 154(5 months) and 278 (9 months)

The following background data will be collected at baseline; age, gender, BMI, ethnicity, marital status, children, educational level, work status, physical work load, leisure time activity, smoking habits, medical history, expectations about treatment effect and characteristics of pain (duration, aggravating factors, morning stiffness, morning pain, relief by NSAIDs, night time pain and former treatment). Emotional distress will be measured using the Hopkins Symptom Checklist-25 [30], fear-avoidance beliefs about physical activity and work with Fear-avoidance beliefs Questionnaire (FABQ) [31]. Subjective health complaints (SHC) will be assessed using a formal inventory that consists of 29 questions concerning severity and duration of subjective somatic and psychological symptoms [32].

At the screening visit a clinical examination including pain provocation tests (springing test, active flexion / extension of the lumbar spine) and neurological tests (strength, toe−/heel walking, sensibility, reflexes, straight leg raising test, reverse Lasegue test)) is performed.

Co-interventions and concomitant medication will be registered at all visits.

### Randomization and blinding

Included patients are allocated in a 1:1 ratio between active treatment and placebo, using a computer randomization procedure stratified by center and previous participation in the Norwegian AIM study [22]. The randomization is blocked within each stratum. Details of block size and allocation sequence generation are provided in a separate document that is unavailable to those who enroll patients or give the treatment. Treatment allocation is performed using the Viedoc™ application. Patients, investigators, treatment administrators, data analyst and statistician are blinded to the treatment allocation. The only unblinded personnel at each site will be the mixing nurse and the controller, who prepares the infusions for treatment administration and control that the correct amount and substance is used. These will have no contact with patients, investigators or treatment administrators other than handing them the prepared infusion bags. Each study site has a site-specific, detailed procedure to ensure blinding during the entire study period. Each site is carefully assessed to ensure that blinding procedures are strictly followed.

Un-blinding of the treatment allocation is only permissible if the safety and well-being of the patient is being compromised. The decision to reveal the treatment allocation during the study may only be done by the principal investigator.

### Trial interventions and schedule

For this study biosimilar infliximab and NaCl used as placebo are defined as Investigational Medicinal Products (IMP). The test treatment is 5 mg/kg infliximab. Both drugs are administered as intravenous infusions. The infusion bags containing the study medication will be prepared by the mixing nurse in identical infusion bags, and applied labels with patient number and dose such that blinding of the participants is secured. The IMPs have the same color and will look the same. After preparing the IMP, the mixing nurse will hand over the IMP to a study nurse, who is blinded to allocation, and authorized by the local principal investigator to administer the infusion. The trial treatment will be given at day 0, 14, 42 and 98, unless unacceptable side effects are encountered. No dose adjustments will be done. All patients take premedication with 1 g of paracetamol and 10 mg of cetirizine prior to each infusion. NaCl is the comparator as there is no proven highly effective pharmacological treatment for cLBP and type 1 MCs [33].

Patients will not receive one particular standard of care prior to inclusion or during the trail. They are allowed to continue their regular LBP therapy, but are encouraged not to start new treatments during the treatment and follow-up period. If a patient needs new treatment during the trial this will be recorded, and the difference in additional therapies between the placebo and intervention group may be assessed.

There will be an end of study visit at day 154 (5 months); the main end-point, and a telephone follow up for safety registration at day 278 (9 months).

### Outcome measures

See Tables 1 and 3 for time points.

#### Primary outcome measure

We will use the Norwegian validated version of the Oswestry Disability Index (ODI) version 2.0 [34, 35]. ODI is a disease-specific disability score recommended for use in LBP research [36]. ODI gives a summed up score from 0 (no disability) to 100 (maximum disability) based on 10 questions.

#### Secondary outcome measures

- Short tau inversion recovery (STIR) signal (intensity and extent) of MCs on MRI

Baseline and follow-up MRI of the lumbar spine includes sagittal T1- and T2-weighted images, axial T2-weighted images and sagittal STIR, fat-water separation and diffusion weighted images. Radiologists will evaluate a range of MC features, including signal intensity and extent.

- Low back pain intensity

Low back pain intensity will be measured as a mean of three Numeric Rating Scale assessments (NRS: 0–10); current LBP, the worst LBP within the last 2 weeks, and usual/mean LBP within the last 2 weeks.

- Roland Morris Disability Questionnaire (RMDQ)

RMDQ is a self-reported disease-specific disability score, ranging from 0 to 24, higher scores indicate more disability [34, 35].

- Health-related quality of life

Quality of life will be measured using EuroQoL-5D-5L (version 2.0), the values are converted to a single utility index, range − 0.59 to 1.0, worse to better respectively [37].

- Co-interventions

Concomitant pharmacological treatment (ATC-coded) and non-pharmacological treatment by self-report.

- Days with sick leave

Patients report the number of days on sick leave last month (if patients are sick listed; degree / % sick listed will also be registered).

- Incidence of adverse events (AEs) and serious adverse events (SAEs) during the study period

AEs and SAEs are registered continuously during the whole study period, and we will assess the frequency, duration and intensity using precise standard medical terminology. In the evaluation, we will also consider serum infliximab concentration and vital signs.

#### Explorative outcome measures

- Leg pain intensity

Patients will be asked to grade the leg pain last week using NRS (0–10).

- Hours with low back pain

Number of days during the last 28 days (4 weeks) the participant had experienced LBP (0–28 days), and, on a typical day, how many hours awake they experienced LBP (0–16 h). The number of days and hours are multiplied (a 0–448 scale).

- Symptom-specific well-being

Measured on a 5-point Likert scale with ‘very satisfied’, ‘some satisfied’, ‘neither satisfied nor dissatisfied’, ‘some dissatisfied’ or ‘very dissatisfied’ [38, 39].

- Patients’ satisfaction

Rated on a 5-point Likert scale; patient’s rate satisfaction with treatment.

- Global perceived effect from baseline

Global Rating of Change is rated on a 7-point Likert scale to quantify a patient’s self-judged improvement from baseline.

- Perceived treatment

Patients are asked which study medicine (Infliximab / placebo / unsure) they think they received during the intervention period, and to what extent infliximab will have an effect on their low back pain.

### Laboratory tests

Hematology, clinical chemistry and acute phase reactants are recorded at all visits. Local laboratories will do the analyses. Serum samples for measurement of infliximab concentrations and anti-drug antibodies will be drawn from all participants at all visits except at screening. All samples will be analyzed at the Department of Medical Biochemistry at Oslo University Hospital, Radiumhospitalet, using in house assays automated on the AutoDELFIA (PerkinElmer, Waltham, MA) immunoassay platform. Results will be recorded in the laboratory data system and transferred to the PI upon the conclusion of the trial. In case of an emergency, serum infliximab and anti-drug levels can be reported to clinicians upon request. Biological samples will be collected and stored in a certified biobank freezer at − 80 °C. The biobank samples will be used for research purposes only, including genetic variation, epigenetics, gene- and protein expression and biomarkers.

Furthermore, patients are asked to collect fecal samples at baseline, 14 weeks and 22 weeks. The patients will receive equipment for collecting fecal samples at home, using Stool Collection Tubes with Stool DNA Stabilizer. The material will be analyzed by sequencing bacterial genome.

### Adverse events and safety

Safety is monitored by the assessment of physical examination and laboratory tests, including hematology, measures of liver and kidney function, and recording adverse events at every visit. Women in childbearing age will take a pregnancy test every month. Each patient is instructed to contact the investigator immediately should they develop symptoms they perceive as serious.

The investigators report all adverse events (AEs) in the eCRF at each visit during the treatment period, post treatment and at safety registration at 9 months. Serious adverse events (SAEs) must be reported to the medical monitor within 24 h after the study site has gained knowledge of the SAE. Any suspected unexpected and serious adverse reaction (SUSAR) will be reported to the Competent Authority according to national regulation. AEs are described in precise medical terminology by the investigator, as well as duration, intensity, attribution, action taken and outcome of the adverse event. A data manager at the Clinical Trial Unit, Oslo University Hospital, will code the AEs and SAEs using the Medical Dictionary for Regulatory Activities (MedDRA.)

The medical monitor keeps detailed records of all SAEs reported by the investigators and performs an evaluation with respect to causality and expectedness.

### Statistical methods and data analysis

The primary objective for this trial is to determine if infliximab improves ODI score from baseline to day 154 (5 months) in patients with cLBP and type 1 MCs, compared to placebo. The null hypothesis is that there is no difference between active treatment and placebo. The alternative hypothesis is that a difference exists.

#### Determination of sample size

The sample size estimation for this study is based on the following assumptions:

- Two-sided test with a 5% significance level
- Power: 80%
- Treatment allocation ratio: 1:1
- Clinically important difference in improvement between groups: 10 ODI points.
- Standard deviation of difference between 154 days/5 months and baseline of 18 ODI points.

With these assumptions, a total sample size of 104 is required (using Stata 16.0 command power twomeans). We added 20% to allow for dropouts, resulting in a total sample size of 126 patients, 63 in each treatment.

#### Data analysis

The following populations will be considered for the analyses:

- Intention to treat population: All randomized participants, regardless of protocol adherence
- Full analysis set: All randomized patients who have taken at least one dose of study medication.
- Safety population: All randomized patients who have taken at least one dose of study medication, i.e. identical to the full analysis set.
- Per Protocol set: All randomized patients who sufficiently comply with the protocol. Criteria for inclusion in the Per Protocol- population will be specified in the statistical analysis plan, and the final criteria will be defined prior to database lock.

#### Planned analyses

The main statistical analysis is planned when the intended number of patients has been included and has either finalized their last assessment or is/has withdrawn according to protocol procedures. Also, all data have to be entered, verified and validated according to the data management plan.

Prior to the statistical analysis, the data base will be locked for further entering or altering of data. A separate statistical analysis plan (SAP) will provide further details on the planned statistical analyses. The SAP will be finalized, signed and dated prior to database lock and published at ClinicalTrials.gov. The treatment allocation will be revealed after the database lock and used in the statistical analysis.

Deviation from the original statistical plan will be described and justified in the reporting of the study. Amendments to the plan can be done until day of database lock.

The primary analyses of the efficacy endpoints will be done in the Full analysis set. Sensitivity analyses will be done in the Per Protocol set.

#### Primary analyses

- Description of data

Continuous variables will be summarized using standard summary statistics such as number of observations (n), mean value, standard deviation (SD), minimum and maximum value, median value, and 1st and 3rd quartiles. Demographic data and other baseline characteristics will be summarized using descriptive statistics.

- Analysis of Efficacy Data

The primary endpoint is the change in the Oswestry Disability Index (ODI) from baseline to day 154 (5 months.) A linear mixed model will be fitted to the primary outcome, including a random intercept and an interaction term of time and treatment. The model will be adjusted for baseline ODI score (prior to treatment) and stratification factors used at randomization. The linear mixed model is equipped to handle missing data.

#### Secondary analyses

Continuous secondary outcomes measured over time will be analyzed in a similar manner as for the primary endpoint. Binary endpoints will be analyzed by logistic regression models, or mixed logistic models, if appropriate.

Further details will be given in the SAP.

### Ethics approval and consent to participate

The BackToBasic study protocol has been approved by the Regional Committee for Medical Research Ethics in Norway (REC South East, reference number 2017/2450) and the Norwegian Medicines Agency (SLV). The study is registered at ClinicalTrials.gov under the identifier NCT03704363. The EudraCT Number: 2017–004861-29. Any important protocol modifications will be reported to the relevant parties.

All patients will receive oral and written information and give their written informed consent before screening. Patients can withdraw their consent at any time. The patient information and informed consent form has been approved by the regional ethics committee before enrolment of patients in the study. The study is conducted in accordance with the Declaration of Helsinki and with ICH/Good Clinical Practice, and will be reported in accordance with the Consolidated Standards of Reporting Trials (CONSORT) guidelines [40]. Registration and storage of patient data are carried out in accordance with international personal data laws (GDPR).

### Trial organization and funding

The BackToBasic study is investigator initiated and independent of the pharmaceutical industry. Oslo University Hospital is sponsor and the Norwegian national program for clinical therapy research, KLINBEFORSK, funds the trial, both are governmental organizations.

## Discussion

This paper presents the design for a randomized, double-blind, placebo-controlled parallel group trial that will assess the effect of infliximab, a TNF-α inhibitor, in patients with low back pain with concomitant type 1 MCs.

The majority of acute episodes with LBP have a good prognosis. However, a significant number of patients experience recurrent episodes or chronic complaints [41, 42]. Current clinical guidelines for non-specific LBP recommend largely generic, symptomatic treatments such as advice to stay active and avoid bed-rest, and analgesic medications, reassurance and exercises. These existing treatments, however, have only small to moderate effects [4, 41]. One explanation for ineffective treatments for non-specific LBP may be that we are unable to direct treatment towards the underlying pathology and instead have to rely upon generic treatments in a heterogeneous patient-population [43].

MCs have been linked to cLBP and suggested as a subgroup within the cLBP [7]. Whatever the etiology of MCs is, it seems clear that local inflammatory responses in the intervertebral disc and vertebral end plates are involved [7, 23]. Data from animal models have shown that nucleus pulposus cells from the disc can be immunogenic and trigger an inflammatory cascade in the vertebra, which results in MCs [20]. Hence, anti-inflammatory treatment in patients with cLBP and MCs could offer a novel treatment strategy.

MCs are strongly associated with adjacent disc degeneration and end plate damage [7]. Tumor necrosis factor (TNF)-α is expressed in both symptomatic disc degeneration and MCs [24]. TNF-α triggers the expression of IL-1β, IL-6, and IL-8, and inhibiting TNF by infliximab reduces their expression in-vitro [44]. Toll-like receptors (TLR TLR1/2/4/6) are expressed in degenerated discs, which can possibly be due to higher TNF levels, driving an autoimmune response [45, 46]. Ohtori et al. found significantly more PGP 9.5-immunoreactive nerve fibres and TNF-immunoreactive cells in the endplates from patients with MCs compared to patients without MCs. They also found more nerve fibres in MC type 1 endplates compared to MC type 2 endplates [25], and concluded that endplate abnormalities are related to inflammation and axon growth induced by TNF. Thus TNF-α could play a central role in the inflammatory responses linked to MCs. Although the pathogenesis of MCs and their role in cLBP is not fully understood, inhibiting TNF-α is a potential treatment-strategy warranting further investigation.

Only patients with type 1 MCs are eligible for this trial. Part of the rationale for this is the higher number of TNF immunoreactive cells found in endplates with type 1 MCs compared to type 2 MCs [25]. Moreover, type 1 MCs are considered to represent a stage of more active inflammation, whereas type 2 and 3 could be a more quiescent stage of the same process [7, 47].

Inhibitors of TNF- α have provided significant treatment advances in several inflammatory diseases, including axial spondyloarthritis [48, 49]. Patients with ankylosing spondylitis treated with infliximab show improvement of inflammation measured by bone marrow edema on STIR sequences [50].

In order to reduce the risk of insufficient dosage, we use the approved dosing regimen for ankylosing spondylitis, which is 5 mg/kg. Serum drug concentration and anti-drug antibody levels are measured before each infusion. Dosages are not adjusted based on serum drug concentration levels, as these are not made available to investigators or treatment administrators. However, serum drug concentration levels and antibody levels can be relevant in the evaluation of potential effects and adverse events, and will be assessed after the trial.

Treatment with infliximab can cause side effects. Inclusion and exclusion criteria have been carefully considered by the study group to ensure that only the patients where immune suppressive treatment is considered safe are enrolled in the trial. Furthermore, treatment is given by trained personnel, patients will be observed for 1 h after the infusions, and participants will be monitored closely for infections or other possible side effects of treatment.

ODI is an approved outcome measure for use in LBP research, especially for evaluation of treatment effect in patients from secondary health care. Based on recommendations we consider a between-group difference of 10 ODI points as clinically relevant, and similar between-group differences have been used in former trials [51–55]. An important change for a patient should be greater than the naturally occurring fluctuations in cLBP. We consider a between-group difference of 10 ODI points to be of importance for the patients, given the nature of the intervention and its associated risks.

In the search of new treatment options for patients with non-specific LBP, there is a need for refined diagnostic assessment to identify possible subgroups that might benefit from specific interventions. This trial will evaluate a range of MC-characteristics by MRI. Machine-learning software will be applied to MRI-data in order to automatically identify MC-features, and hence improve the assessment of MCs. Along with the clinical trial we will also explore potential biomarkers, including gene expression and epigenetic profiling, to increase our knowledge of underlying factors of MC-related LBP. A full set of pre-specified hypotheses is available in the Appendix (Table 4).

Participant recruitment was initiated December 2018 and is ongoing. Anticipated recruitment period is 3 years. Upon study completion the results of the trial will be submitted for publication in a publicly accessible database of clinical study results. Also the results will be submitted to the Competent Authority and the Ethics Committee according to EU and national regulations.

## Data Availability

Not applicable.

## Appendix

**Table 4:** List of pre specified objectives

	Objectives	Endpoints
Primary	Objective A:	Primary efficacy endpoint:
	To evaluate the effect of biosimilar infliximab on disease specific disability in patients with chronic low back pain and Modic changes type 1	Oswestry Disability Index change from baseline to day 154 (5 months)
Secondary	Objective B:	Secondary endpoints:
	To evaluate the effect of biosimilar infliximab on bone marrow oedema in patients with chronic low back pain and Modic changes type 1	– Short tau inversion recovery (STIR) signal (intensity and extent) of MCs from baseline to 5/6 months
	Objective C:	Secondary endpoints:
	To evaluate the effect of biosimilar infliximab on pain in patients with chronic low back pain and Modic changes type 1	– LBP intensity at day 154 (5 months) follow-up
	Objective D:	Secondary endpoints:
	To evaluate the effect of biosimilar infliximab on disease specific disability in patients with chronic low back pain and Modic changes type 1	– Roland Morris Disability Questionnaire at day 154 (5 months) follow-up
	Objective E:	Secondary endpoints:
	To evaluate whether the short tau inversion recovery (STIR) signal (intensity and extent) of Modic changes type 1 on baseline MRI predicts clinical outcome	– ODI score at day 154 (5 months) follow-up
		– LBP intensity at day 154 (5 months) follow-up
	Objective F:	Secondary endpoints:
	To evaluate the effect of biosimilar infliximab on health-related quality of life in patients with chronic low back pain and Modic changes type 1	– Health-related quality of life (the EQ-5D) at day 154 (5 months) follow-up
	Objective G:	Secondary endpoints:
	To evaluate cost-effectiveness of biosimilar infliximab in patients with chronic low back pain and Modic changes type 1	– ODI score at day 154 (5 months) follow-up
		– Health-related quality of life (the EQ-5D) at day 154 (5 months) follow-up
		– Co-interventions (pharm. and non-pharmacological)
		– Days with sick-leave
	Objective H:	–
	To evaluate the incidence of AEs and SAEs in patients who receive biosimilar infliximab	
Exploratory	To evaluate the effect of biosimilar infliximab versus placebo on other outcome measures not mentioned above in patients with chronic low back pain and Modic changes type 1	Exploratory endpoints at day 154 (5 months) follow-up:
		– Leg pain intensity
		– Hours with LBP during the last 4 weeks
		– Symptom-specific well-being
		– Days with sick leave
		– Co-interventions
		– Patients’ satisfaction
		– Global perceived effect
	To evaluate the long-term effect of biosimilar infliximab versus placebo on pain and disability in patients with chronic low back pain and Modic changes type 1	Exploratory endpoints:
		– ODI at 9 months follow-up
		– Leg pain intensity at 9 months follow-up
	To evaluate the feasibility of machine learning to assess imaging features of Modic changes type 1	Exploratory endpoints:
		– Machine detected features of MCs baseline and 6 months
	To investigate the underlying biological mechanisms of Modic changes type 1 through combined gene expression and epigenetic profiling and correlate with clinical outcome	Exploratory endpoints:
		– Epigenetic patterns, longitudinal gene- and protein expression, genetic variation at baseline, end of treatment and at 5 months
	To identify biomarkers for any TNF-alfa blockers response in patients with chronic low back pain and Modic changes type 1	Exploratory endpoints:
		– Protein expression at baseline

## Abbreviations

LBP: Low back pain
cLBP: Chronic LBP
MCs: Modic changes
MRI: Magnetic Resonance Imaging
RCT: Randomized Controlled Trial
NRS: Numerical Rating Scale
ODI: Oswestry Disability Index
GCP: Good Clinical Practice
PROMs: Patient Reported Outcome Measures
FABQ: Fear-avoidance beliefs Questionnaire
SHC: Subjective Health Complaints
IMP: Investigational Medicinal Products
STIR: Short tau inversion recovery signal,
RMDQ: Roland Morris Disability Questionnaire
AEs: Adverse Events
SAEs: Serious adverse events
SUSAR: Suspected Unexpected Serious Adverse Reaction
MedDRA: Medical Dictionary for Regulatory Activities
SAP: Statistical Analysis Plan
SD: Standard deviation
CONSORT: Consolidated Standards of Reporting Trials
